# Freehand S2‐Alar‐Iliac Screw Placement Technique in Lumbosacral Spinal Tumors: A Preliminary Study

**DOI:** 10.1111/os.13434

**Published:** 2022-08-16

**Authors:** Wending Huang, Lun Xu, Weiluo Cai, Mo Cheng, Zhengwang Sun, Shengping Wang, Wangjun Yan

**Affiliations:** ^1^ Department of Musculoskeletal Oncology, Shanghai Cancer Center Fudan University Shanghai China; ^2^ Department of Oncology, Shanghai Medical College Fudan University Shanghai China; ^3^ Department of Radiology, Shanghai Cancer Center Fudan University

**Keywords:** freehand, lumbosacral fixation, lumbosacral spine, S2‐alar‐iliac screw, tumor

## Abstract

**Objective:**

S2‐alar‐iliac (S2AI) screw technique is widely used in spinal surgery, but it is rarely seen in the field of spinal tumors. The aim of the study is to report the preliminary outcomes of the freehand S2AI screw fixation after lumbosaral tumor resection.

**Methods:**

The records of patients with lumbosacral tumor who underwent S2AI screw fixation between November 2016 to November 2020 at our center were reviewed retrospectively. Outcome measures included operative time, blood loss, complications, accuracy of screws, screw breach, and overall survival. Mean ± standard deviation or range was used to present continuous variables. Kaplan–Meier curve was used to present postoperative survival.

**Results:**

A total of 23 patients were identified in this study, including 12 males and 11 females, with an average age of 47.3 ± 14.5 (range,15–73). The mean operation time was 224.6 ± 54.1 (range, 155–370 min). The average estimated blood loss was 1560.9 ± 887.0 (600–4000 ml). A total of 46 S2AI screws were implanted by freehand technique. CT scans showed three (6.5%) screws had penetrated the iliac cortex, indicating 93.5% implantation accuracy rate. No complications of iatrogenic neurovascular or visceral structure were observed. The average follow‐up time was 31.6 ± 15.3 months (range, 13–60 months). Two patients' postoperative plain radiography showed lucent zone around the screw. One patient underwent reoperation for wound delayed infection. At the latest follow‐up, eight patients had tumor‐free survival, 11 had survival with tumor, and four died of disease.

**Conclusion:**

The freehand S2AI screw technique is reproducible, safe, and reliable in the management of lumbosacral spinal tumors.

## Graphical abstract

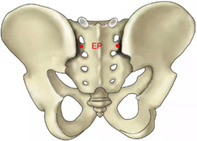

## Introduction

Spinopelvic fixation is indicated in various diseases, including kyphoscoliosis in adults, severe spondylolisthesis, severe pelvic obliquity, and sacral fractures with pelvic diastasis^1^, ^2^, ^3^. Over the years, a variety of techniques have been used for lumbopelvic fixation, including Galveston iliac rods, Jackson intrasacral rods, the Kostuik transiliac bar, iliac screws, S1 and S2 pedicle screws, and S2‐alar‐iliac (S2AI) screws^2^, ^3^, ^4^. At present, iliac screws and S2AI screws have been the predominant methods for lumbopelvic fixation, providing solid bony fusion across the lumbosacral junction.

The S2AI screw technique, first proposed by Sponseller^5^ in 2007, has become a popular method for spinopelvic fixation over the past decade^1^, ^2^, ^4^, ^6^. This technique is mainly used for long‐segment spinal fusion to the sacrum in children and adults with a spinal deformity or high‐grade spondylolisthesis. Despite its ubiquitous use, the application of this technique in spinal reconstruction after resection of lumbosacral spinal tumors has been rarely reported. Spinal reconstruction following lumbosacral spinal tumor resection has been a great challenge. Pedicle screws are usually placed for fusion extending to S1 or S2 after lumbosacral spinal tumor resection. Although the combination of S1 and S2 pedicle screws is stronger compared to S1 screws alone, the biomechanical strength is not satisfactory because there is no increase in the overall strength of the lumbosacral fixation construct^7^, ^8^.

The advent of the S2AI technique provides an alternative method to overcome the above problems. Biomechanical studies have shown that S2AI screws have the same biomechanical strength as iliac screws and can be used as an alternative to iliac screws^9^, ^10^, ^11^, ^12^. Nowadays, S2AI screws have been widely used in spine surgery. Meanwhile, the relative shallow learning curve of the S2AI screw makes the freehand technique popular^9^, ^13^, ^14^, ^15^, ^16^. However, the application of the technique in lumbosacral spinal tumors has rarely been reported.

Our institutional senior surgeons prefer a freehand technique of S2AI according to the anatomical landmarks. The purposes of the study were: (1) to investigate the feasibility of the freehand S2AI screw technique in lumbosacral spinal tumors; (2) to reveal the complications and clinical outcomes of S2AI screw fixation.

## Materials and Methods

### Study Design

This study was approved by the institutional review board (IRB/IEC: 2010230–2), and all patients provided informed consent. Patients with lumbosacral spinal tumors who underwent tumor resection and spinal‐pelvic reconstruction between November 2016 and November 2020 were reviewed. The inclusion criteria were as follows: (1) patients with lumbosacral spinal tumor needing spinal‐pelvic reconstruction, (2) the bone between the dorsal foramina of S1–2 was not invaded by the tumor, (3) patients with complete data and follow‐up for more than 12 months. The exclusion criteria were as follows: (1) patients with deformity, degeneration, trauma, or infection; (2) those without S2AI screw implantation. All the S2AI screws were placed by senior spine surgeons.

### Preoperative Evaluation

All patients in our series were evaluated meticulously by our group after admission. All patients preoperatively underwent X‐ray, computed tomography (CT) with three‐dimension reconstruction, and magnetic resonance imaging (MRI). Patients with metastatic spinal tumors were examined by positron‐emission tomography/computed tomography (PET/CT) or single‐photon emission computed tomography (SPECT) scan. Based on CT scans, detailed screw implantation plans were drafted, including entry point, trajectory direction, and screw length.

### Surgical Technique

**Fig. 1:**
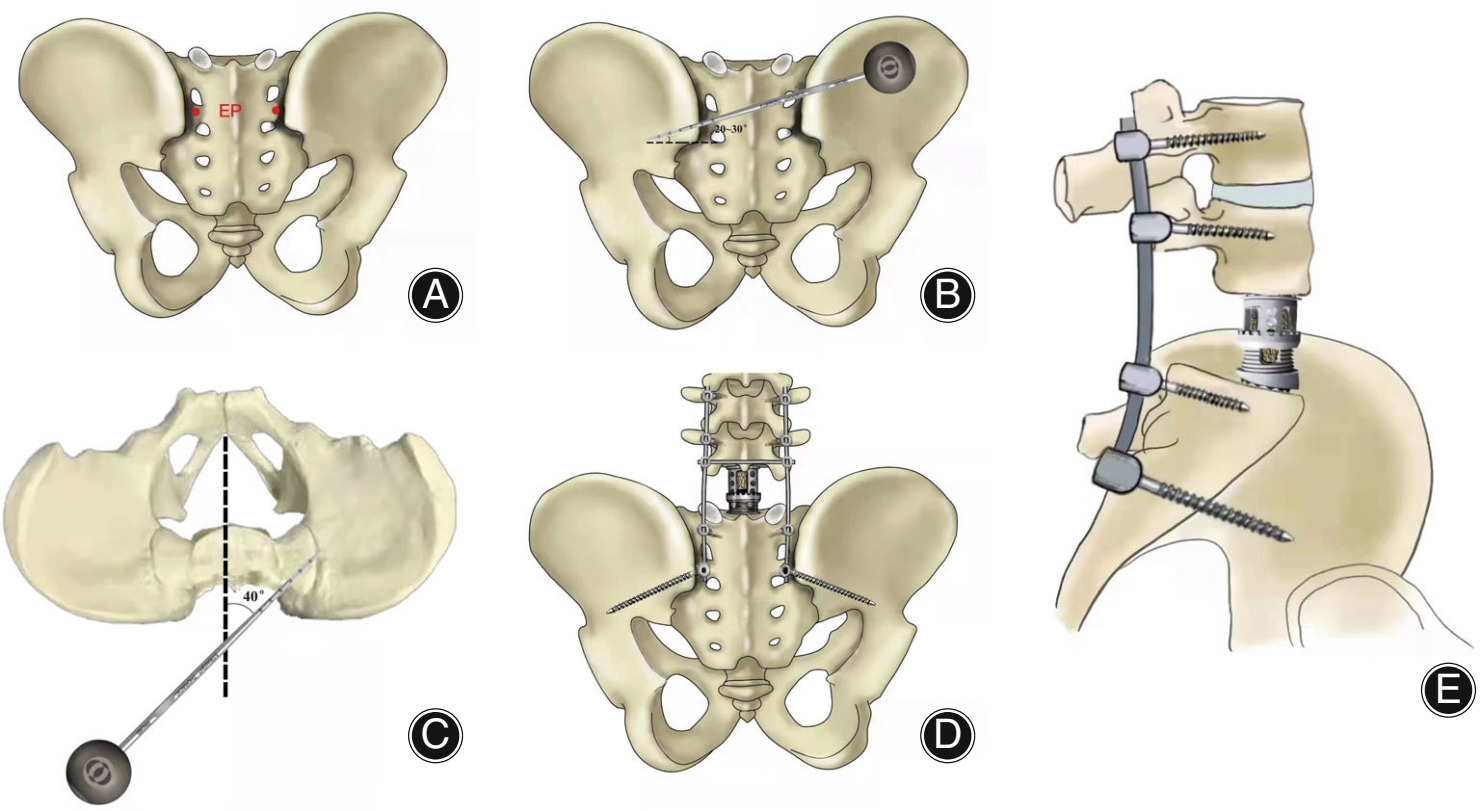
(A) Entry point (EP) of S2AI screw is 2 mm lateral to the midpoint between the S1 and S2 dorsal foramen. The trajectory direction was 20°–30° caudally in the sagittal plane (B) and approximately 40° horizontally in the axial plane (C), pointing to the anterior inferior iliac spine (AIIS). (D) Anteroposterior diagram of the postoperative reconstruction of lumbo‐pelvis with S2AI screws after tumor resection. (E) Lateral diagram showed the sagittal effect after lumbopelvic reconstruction

After general anesthesia, the patient was placed in the prone position. A posterior midline incision was made, and meticulous subperiosteal dissection of the posterior elements was performed to extend to the sacroiliac joint laterally. Then S1 and S2 dorsal foramen were confirmed. The S2AI screw placement was performed by using the anatomic landmarks. The entry point was 2 mm lateral to the midpoint between the S1 and S2 dorsal foramen (Figure 1A)^9^, ^10^. The screw trajectory direction was 20°–30° caudally in the sagittal plane and approximately 40° horizontally in the axial plane, pointing to the anterior inferior iliac spine (AIIS), where roughly two fingers of the superior border of the greater trochanter of the femur and can be palpated intraoperatively (Figure 1B,C). The entry point was drilled to make a 5‐mm‐deep cortical breach by a high‐speed burr. A sharp pedicle probe was advanced toward the sacroiliac joint at the above angle. When the probe reached the sacroiliac joint, at an approximate distance of 35 mm as described by Chang *et al*.^6^, an increased resistance was experienced. A ball‐tip probe was used to palpate the osseous bottom of the channel. Then the pedicle probe was advanced toward the AIIS until it entered the ilium at a depth of about 80 mm. A ball‐tip was reinserted to palpate to ensure that the floor and walls of the screw trajectory were intraosseous. If soft tissue or sudden advancement was palpated, a cortical breach was identified, and the screw path was salvaged by redirecting the pedicle probe in a more appropriate direction. The ball‐tip probe was removed, and the screw length reconfirmed with a hemostat clamp. The S2AI trajectory was undertapped 1 mm less than the desired screw diameter. Finally, a screw in a diameter of 7.0–8.5 mm and a length of 80–90 mm was inserted according to the measurement. The operation was completed according to the preoperative plan (Figure 1D,E).

### Follow‐Up and Evaluation

Data on patient demographics, tumor site, pathology, operation record, radiographic outcomes, and complications were collected and reviewed. Patients with metastatic tumors were followed up every 3 months after surgery. Those with primary lesions were routinely followed up every 3 months in the first 2 years and semiannually after that. Adjuvant therapies were added depending on the type of pathology. The accuracy of the S2AI screws was assessed by postoperative plain radiography and CT scans. All CT scans were reviewed independently by a senior radiologist for radiographic outcomes. When a cortical breach was found, the breach direction and distance were recorded and measured. Breaches were classified into four grades according to the severity: grade 0 (no breach), grade 1 (a breach distance of less 3 mm, mild), grade 2 (3–6 mm, moderate), and grade 3 (more than 6 mm)^17^.

### Statistical Analysis

All statistical analyses were carried out using SPSS 22.0 (IBM Corp.). Continuous variables were presented as the mean ± standard deviation. The Kaplan–Meier method was used to estimate postoperative survival, and survival curves were analyzed and presented.

## Results

### General Data

General data of patients were summarized in Table 1. A total of 23 patients were included in this study, including 12 males and 11 females, with an average age of 47.3 ± 14.5 (range: 15–73). All tumors were located in the lumbosacral spine region, including two in L4–5, seven in L5, six in L5‐S1, eight in S1. Patients' surgical and follow‐up data were summarized in Table 2. The mean operation time was 224.6 ± 54.1 min (range: 155–370 min). The average estimated blood loss was 1560.9 ± 887.0 ml, with a range of 600–4000 ml.

**TABLE 1:** Summary data of 23 patients with at least 12‐month follow‐up after S2AI screw placement

No.	Sex	Age	Location	Diagnosis	Revision surgery	ATBS	WBB staging	Surgical strategy	PE
1	M	73	L5	Lung cancer	No	Target	4–8, A–C	Gross total resection	No
2	F	47	S1	Breast cancer	No	CT + ET + diphosphonate	5–8, B–D	Separation surgery	No
3	M	64	L5	Renal carcinoma	No	Target+ diphosphonate	5–8, B–D	Gross total resection	Yes
4	F	41	L5‐S1	Breast cancer	No	CT + ET + diphosphonate	4–6, A–C	Separation surgery	No
5	F	52	L5	Breast cancer	No	CT + ET + RT + diphosphonate	5–8, A–C	Gross total resection	No
6	F	50	S1	Lung cancer	No	Target	6–10, A–D	Piecemeal resection	No
7	M	54	L5	Lung cancer	No	CT + diphosphonate	5–8, B–D	Gross total resection	No
8	M	59	S1	Hepatocarcinoma	No	Target	4–7, A–D	Piecemeal resection	Yes
9	F	58	S1	Rectal cancer	No	CT + Target + RT + diphosphonate	4–8, A–D	Piecemeal resection	No
10	F	60	L5‐S1	Breast cancer	No	CT + ET + diphosphonate	5–10, B–D	Piecemeal resection	No
11	F	38	L5	Cervical cancer	No	CT + diphosphonate	6–9, A–C	Piecemeal resection	No
12	M	53	S1	Renal carcinoma	No	No	3–5, B–C	Piecemeal resection	No
13	M	54	S1	Hepatocarcinoma	No	No	7–10, A–D	Piecemeal resection	Yes
14	F	30	L5	GCTB	Yes	Denosumab	5–8, A–D	Gross total resection	Yes
15	F	30	L5	GCTB	No	Denosumab	6–9, A–D	En bloc resection	Yes
16	F	51	S1	Chondrosarcoma	No	No	4–5, B–C	En bloc resection	No
17	M	22	L4–5	Synovial sarcoma	No	CT	3–8, A–D	Gross total resection	Yes
18	M	30	S1	Chordoma	No	No	5–8, B–D	En bloc resection	No
19	M	64	L5‐S1	Schwannoma	No	No	4–6, A–D	En bloc resection	No
20	M	33	S1	Paraganglioma	No	No	5–8, A–D	Gross total resection	No
21	F	55	L5	LCH	No	No	4–8, B–D	En bloc resection	No
22	M	15	L4–5	Ewing sarcoma	Yes	CT	4–7, A–D	Gross total resection	Yes
23	M	55	L5‐S1	SFT	No	No	5–9, A–D	Gross total resection	Yes

Abbreviations: ATBS, Adjuvant therapy before surgery; CT, chemotherapy; ET, Endocrine therapy; LCH, Langerhans cell histiocytosis; PE, preoperative embolization；GCTB, giant cell tumor of bone; RT, radiotherapy; SFT, solitary fibrous tumor.

**TABLE 2:** Surgical data and outcomes of patients with at least 12‐month follow‐up after S2AI screw placement

No.	OT (mins)	BL (ml)	SL (mm)	SD (mm)	Screw Breach	Reconstruction	Complications	Adjuvant therapy	FU/outcomes
1	210	1400	80	7.0	No	L3–4, S1 PS + S2AI + AVB + BC		Target+ diphosphonate	31/DOD
2	170	800	80	7.0	No	L4–5 PS + S2AI		ET + diphosphonate	34/SWT
3	260	1500	80	7.0	No	L3–4, S1 PS + S2AI + AVB + AB	Cerebrospinal fluid leak	Target+ diphosphonate	60/TFS
4	190	800	80	7.5	No	L3–5 PS + S2AI		ET + diphosphonate	29/SWT
5	250	1800	80	7.0	Right (grade 1)	L3–4, S1 PS + S2AI+ TM + BC		ET + diphosphonate	56/SWT
6	175	1300	80	7.0	No	L4–5 PS + S2AI	Screw lucent zone	Target+ diphosphonate	36/SWT
7	265	1400	90	8.5	No	L3–4, S1 PS + S2AI + AVB + BC		CT + RT+ diphosphonate	14/DOD
8	155	1900	90	8.5	No	L4–5 PS + S2AI		Target+RT+ diphosphonate	21/SWT
9	190	600	80	7.0	No	L4–5 PS + S2AI	Wound infection	Target+ diphosphonate	13/DOD
10	210	1000	80	7.0	No	L3–4, S1 PS + S2AI + AVB + BC		ET + RT + diphosphonate	20/SWT
11	195	1100	80	8.5	No	L3–4, S1 PS + S2AI + AVB + BC		RT + diphosphonate	19/SWT
12	180	4000	80	8.5	No	L4–5 PS + S2AI		Target+PD1 + diphosphonate	15/SWT
13	170	1500	90	8.5	No	L4–5 PS + S2AI	Cerebrospinal fluid leak	Target+diphosphonate	15/SWT
14	290	3200	80	7.0	Right (grade 2)	L3–4, S1 PS + S2AI + AVB + AB		Denosumab	52/TFS
15	275	1600	80	7.0	No	L2–4, S1 PS + S2AI + AVB + AB		Denosumab	24/TFS
16	205	700	80	7.0	Left (grade 1)	L4–5 PS + S2AI		No	26/TFS
17	370	2200	80	7.0	No	L3–4, S1 PS + S2AI + AVB + AB		CT + RT	32/TFS
18	210	900	90	8.5	No	L4–5 PS + S2AI		RT	44/TFS
19	190	1200	80	7.5	No	L4–5 PS + S2AI	Delayed wound infection	No	27/TFS
20	160	600	80	7.5	No	L4–5 PS + S2AI		RT	26/SWT
21	270	1200	80	7.0	No	L3–4, S1 PS + S2AI + AVB + AB	Cerebrospinal fluid leak	No	56/TFS
22	310	1700	80	7.0	No	L2–3, S1 PS + S2AI + AVB + AB	Screw lucent zone	CT + RT	60/SWT
23	265	3500	80	7.5	No	L3–4, S1 PS + S2AI + TM + BC	Lung infection	Target (Pazopanib) + RT	16/DOD

Abbreviations: AB, autogenous/allogeneic bone; AVB, artificial vertebral body; BC, bone cement; CT, chemotherapy; DOD, died of disease; ET, Endocrine therapy; FU, Follow‐up; OT, operation time; PD1: programmed cell death protein‐1 inhibitor; PS, pedicle screws; RT, radiotherapy; SD, screw diameter; SL, Screw length; SWT, survival with tumor; TFS, tumor free survival; TM, titanium mesh.

### Surgery Outcomes and Radiographic Evaluation

**Fig. 2:**
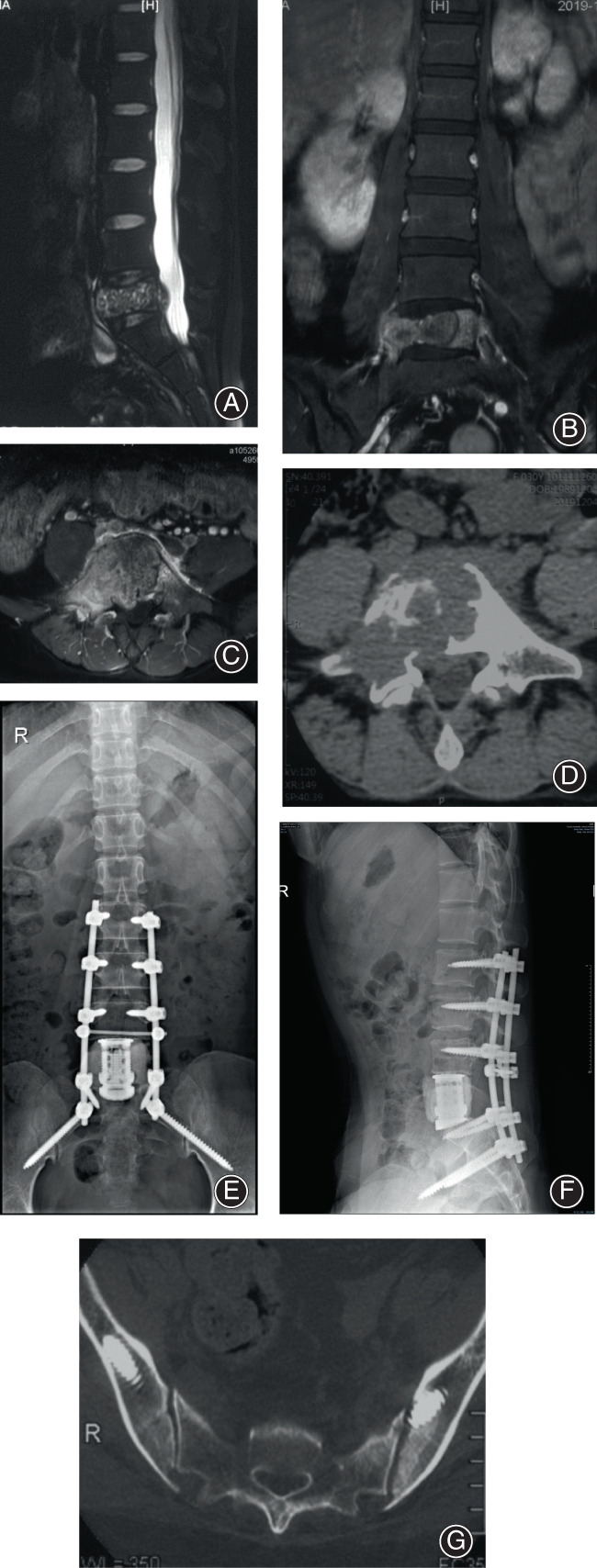
Case 15. Giant cell tumor of bone at L5 in a 30‐year‐old woman. (A) Sagittal T2‐weighted magnetic resonance imaging showed the tumor involving L5 vertebral body. Coronal (B) and axial (C) T1‐weighted enhanced magnetic resonance imaging demonstrated the extent of the tumor with spinal canal compromise. (D) Axial computed tomographic scan demonstrated the tumor with osteolytic destruction. Anteroposterior (E) and lateral (F) radiographs showed a stable construct at 2 years postoperatively. (G) Postoperative computed tomographic scan demonstrated no breach of the screws

**Fig. 3:**
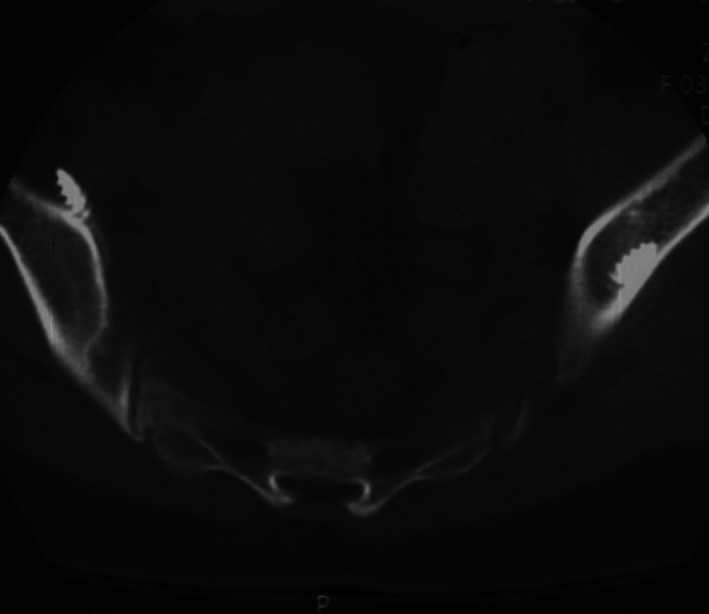
Postoperative computed tomographic scan on axial slice showed an anterior breach of S2AI screw on the right side

A total of 46 S2AI screws were implanted successfully by senior spine surgeons. All screws were inserted successfully without replacement (Figure 2). The length of implanted screws was detailed in Table 2. All patients received postoperative plain radiography and CT scans to evaluate the locations of the screws. The results confirmed that 43 screws were in good positions with an accuracy rate of 93.5% (43/46). Three screw breaches (6.5%) were observed in three patients, including one screw penetrating the anterior iliac cortex and two screws penetrating posterior iliac cortexes (Figure 3). Two of the screws were graded as 1 (mild), and one was graded as 2 (moderate).

### Complications

All surgery‐related complications were detailed in Table 2. The three patients with screw breaches showed no complications of the vessel or visceral injuries. Eight (34.8%) of the patients experienced postoperative complications. Three (13%) patients had a postoperative cerebrospinal fluid leak. Two (8.7%, case 9, 19) patients had wound infection and underwent reoperation. One patient (case 9) had a treatment history of target therapy and radiotherapy before surgery. Another one developed delayed infection 7 months after surgery and underwent a debridement procedure. Two (8.7%) patients had evidence of S2AI screw lucent zones, however, no fixation failure occurred. One patient developed a pulmonary infection and recovered after symptomatic treatment.

### Follow‐Ups

**Fig. 4:**
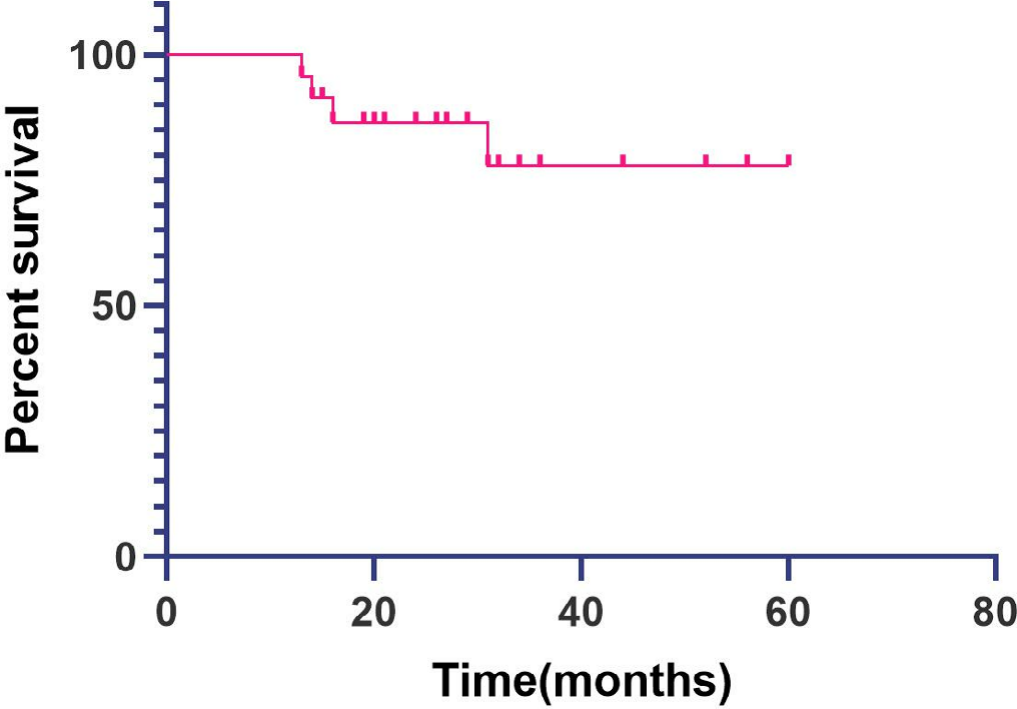
Kaplan–Meier survival curve

The mean follow‐up was 31.6 ± 15.3 months (range, 13–60 months) after surgery. None of the patients had implant prominence or pain during the follow‐up. Twenty patients received systemic adjuvant therapy according to the type of pathology, including chemotherapy, endocrine therapy, target therapy, immunotherapy, radiotherapy, bisphosphonates, and denosumab. The other three patients (case 16, 19, 21) who underwent en bloc resection of the tumor were regularly followed up only after surgery. At the latest follow‐up, eight patients had tumor‐free survival, 11 survived with tumor, and four died of the disease. The Kaplan–Meier survival curve demonstrated an overall survival rate of 82.6% during a mean follow‐up time of 31.6 months (Figure 4).

## Discussion

In this study, all screws were successfully placed by freehand. The results showed three (6.5%) screws penetrated the iliac cortex, indicating a 93.5% of implantation accuracy rate. No complications of iatrogenic neurovascular or visceral structure were observed.

### Feasibility and Safety of S2AI Screw

The S2AI screw technique for spinopelvic fixation has been described in detail in the literature. This technique can be performed with the assistance of a navigation system, robot, C‐arm fluoroscopy, or freehand placement^9^, ^13^, ^18^, ^19^, ^20^, ^21^, ^22^, ^23^. However, due to navigation or robotic system not being available in all centers, it was challenging to popularize and promote the technique. Furthermore, the requirement of intraoperative CT scan for the navigation system or robotic assist increases radiation exposure. The freehand S2AI technique, which was guided by anatomical landmarks, was presented and described in detail by Park *et al*.^13^ in 2015. Studies have shown that freehand technique based on anatomical landmarks has become a mature and consistent technique^9^, ^13^, ^14^, ^15^, ^16^, ^18^, ^24^, ^25^. After mastering S2AI screw technique, we began to use the freehand technique in 2016.

Previous anatomic and clinical studies have demonstrated that the freehand S2AI screw technique is as safe, accurate, and reliable as navigation and robotics. Park *et al*. described a freehand S2AI screw technique in fresh‐frozen human cadavers using pelvic anatomic landmarks^13^. Eight screws were implanted with the direction of an approximately 20° caudal angle in the sagittal plane and 30° horizontal angle in the coronal plane connecting the posterior superior iliac spine (PSIS). They reported an accuracy rate of 100% evaluated by fluoroscopy and naked eye examination. Their team had also reported a total of 45 S2AI screws in 23 patients, only five of which demonstrated a breach, with no visceral or neurovascular complications^14^. Lombardi and colleagues preferred the freehand technique when spinopelvic fixation was required, which was thought to be a simple, safe, and effective method^2^. Shillingford *et al*. described the freehand S2AI screw technique in which the entry point is lateral to the midpoint of the S1–2 dorsal foramen, directed toward the AIIS by aiming to a point just cephalad to the posterior edge of the PSIS and perpendicular to the lateral sacral crest^9^. The results showed that the average caudal angle was 24.2° ± 10.0° in the sagittal plane, and the mean horizontal angle was 39.3° ± 8.2° in the axial plane. The reported accuracy was 95% and only 5% of the screws were placed with cortical breaches. Their team then compared the accuracy of the freehand technique with that of the robot‐guided insertion of S2AI screws, showing no difference in accuracy between the two methods (94.9% *vs*. 97.8%, *p* = .630)^25^.

In our series, individualized protocols were performed to place S2AI screws. We excluded patients with tumors involving the bone of the dorsal foramina between S1 and S2 due to the compromised anatomic landmark of the entry point. The entry point was 2 mm lateral to the midpoint of the dorsal foramen of S1–S2. The screw direction was 20°–30° caudally in the sagittal plane and 40° horizontally in the axial plane, pointing to the AIIS, about two fingers of the superior border of the greater trochanter. The postoperative evaluation showed that only three (6.5%) screws were demonstrated to have cortex breaches, and the accuracy rate of screw placement was 93.5%. There were no neurovascular and visceral injury complications related to S2AI screws during the operations, which was consistent with reports in the literature.

### Advantages and Disadvantages of S2AI Screw

The advantages of S2AI screws for spinopelvic fixation make this technique more popular in recent decade. Most importantly, S2AI screw placement requires less dissection of the soft tissue. The rate of wound infection was significantly lower in patients with S2AI screws compared with those with iliac screws because the iliac screw technique requires dissection of the subcutaneous tissue off the lumbosacral fascia to the level of the PSIS^26^, ^27^, ^28^, ^29^, ^30^, ^31^, ^32^, ^33^, ^34^. In De la Garza Ramos's meta‐analysis, the infection rate in the iliac screw group was 25.4% compared with only 2.6% in the S2AI group^34^. In our series, the wound infection was 8.7%, which was similar to the literature reports. Secondly, the location of conventional iliac screws is not in line with proximal lumbar screws, requiring offset‐connectors for the connection of rod‐system and iliac screws. In contrast to iliac screws, S2AI screws are in line with the posterior rod‐system, without requiring connectors or complex bends for the connection with proximal lumbar screws. Furthermore, due to the more extensive soft tissue dissection, iliac screw implantation causes more soft tissue damage than S2AI screws. Moreover, the deeper location of S2AI screws entry point and more extensive soft tissue covering than conventional iliac screws results in less risk of implant prominence, reducing associated complications.

However, there are also disadvantages to S2AI screw fixation. Some scholars believe that S2AI screw fixation has a higher rate of implant failure. Guler *et al*. found a failure rate of 35% for S2AI screws and 12% for iliac screws (*p* > .05) in their retrospective study^35^. All screw breakages were associated with the S2AI technique. Therefore, long‐term follow‐up results of S2AI screws need to be supported by large sample studies. There was no failure of internal fixation in our series during the follow‐up. One of the reasons was that no patients had a spinal deformity, and the balance between sagittal and coronal planes was not disturbed after the operation.

### Indications

Current indications for spinopelvic fixation with S2AI screws mainly include high‐grade spondylolisthesis, long‐segment fusion constructs, flat back deformities, three‐column osteotomies, and correction of pelvic obliquity^2^, ^3^, ^4^. However, there is no consensus on the indications in the literature. It has been reported that S2AI screw fixation is also suitable for sacropelvic reconstruction after sacral tumor resection^2^, ^30^, ^36^, but it is not widely used because lumbosacral spinal tumor is not common.

### Strengths and Limitations

To the best of our knowledge, this study has the largest group of patients with lumbosacral tumors treated with the S2AI technique. Tumors in this region often require segmental resection or spondylectomy, which can cause spinal instability and require three‐column reconstruction. In order to minimize surgical complications, S2AI screw fixation was selected as the preferred method. Therefore, we posit that the S2AI technique is suitable for spinopelvic reconstruction when no tumor is present in the bone between the S1–S2 dorsal foramina.

This study has limitations. First, this is a retrospective study design without a control group. Second, the number of included patients was small. Third, the group of patients was heterogeneous in pathological diagnosis, which may lead to bias in the sample. Fourth, the follow‐up time was not long enough to obtain extensive clinical data. Therefore, further prospective controlled studies with a large sample and long‐term follow‐up are required.

### Conclusion

The freehand S2AI screw technique is reproducible, safe, and reliable in the management of lumbosacral spinal tumors. It is worth popularizing because this technique can decrease soft tissue dissection, potentially reduce operative time, intraoperative fluoroscopy and radiation exposure, and yield fewer wound complications.

## AUTHORS' CONTRIBUTIONS

Wending Huang, Lun Xu conceptualized, collected, and interpreted the clinical data, and wrote the manuscript. Wangjun Yan, Wending Huang contributed to design of the work and revised the manuscript critically for important intellectual content. Weiluo Cai, Mo Cheng, Zhengwang Sun, Shengping Wang contributed to data acquisition and revised the manuscript. All authors read and approved the final manuscript.

## CONFLICTS OF INTEREST

There were no conflicts of interest in this study.

## FUNDING INFORMATION

No funds were received in support of this work.

## ETHICAL APPROVAL

The study was approved by the Ethics Committee of Shanghai Cancer Center.

## References

[1] Kebaish KM . Sacropelvic fixation: techniques and complications. Spine. 2010;35:2245–51. 21102300 10.1097/BRS.0b013e3181f5cfae

[2] Lombardi JM , Shillingford JN , Lenke LG , Lehman RA . Sacropelvic fixation: when, why, how? Neurosurg Clin N Am. 2018;29:389–97. 29933806 10.1016/j.nec.2018.02.001

[3] Moshirfar A , Rand FF , Sponseller PD , et al. Pelvic fixation in spine surgery. Historical overview, indications, biomechanical relevance, and current techniques. J Bone Joint Surg Am. 2005;87:89–106. 16326728 10.2106/JBJS.E.00453

[4] Jain A , Hassanzadeh H , Strike SA , Menga EN , Sponseller PD , Kebaish KM . Pelvic fixation in adult and pediatric spine surgery: historical perspective, indications, and techniques. J Bone Joint Surg Am. 2015;97:1521–8. 26378268 10.2106/JBJS.O.00576

[5] Sponseller PD . The S2 portal to the ilium. Semin Spine Surg. 2007;2:83–7.

[6] Chang TL , Sponseller PD , Kebaish KM , Fishman EK . Low profile pelvic fixation: anatomic parameters for sacral alar‐iliac fixation versus traditional iliac fixation. Spine. 2009;34:436–40. 19247163 10.1097/BRS.0b013e318194128c

[7] Zindrick MR , Wiltse LL , Widell EH , et al. A biomechanical study of intrapeduncular screw fixation in the lumbosacral spine. Clin Orthop Relat Res. 1986;203:99–112. 3956001

[8] Galbusera F , Casaroli G , Chande R , Lindsey D , Villa T , Yerby S , et al. Biomechanics of sacropelvic fixation: a comprehensive finite element comparison of three techniques. Eur Spine J. 2020;29:295–305. 31773275 10.1007/s00586-019-06225-5

[9] Shillingford JN , Laratta JL , Tan LA , Sarpong NO , Lin JD , Fischer CR , et al. The free‐hand technique for S2‐alar‐iliac screw placement: a safe and effective method for sacropelvic fixation in adult spinal deformity. J Bone Joint Surg Am. 2018;100:334–42. 29462037 10.2106/JBJS.17.00052

[10] Hoernschemeyer DG , Pashuck TD , Pfeiffer FM . Analysis of the S2 alar‐iliac screw as compared with the traditional iliac screw: does it increase stability with sacroiliac fixation of the spine? Spine J. 2017;17:875–9. 28185981 10.1016/j.spinee.2017.02.001

[11] JR OB , Yu W , Kaufman BE , et al. Biomechanical evaluation of S2 alar‐iliac screws effect of length and quad‐cortical purchase as compared with iliac fixation. Spine. 2013;38:E1250–5. 23759811 10.1097/BRS.0b013e31829e17ff

[12] Burns CB , Dua K , Trasolini NA , Komatsu DE , Barsi JM . Biomechanical comparison of spinopelvic fixation constructs: iliac screw versus S2‐alar‐iliac screw. Spine Deform. 2016;4:10–5. 27852493 10.1016/j.jspd.2015.07.008

[13] Park JH , Hyun SJ , Kim KJ , Jahng TA . Free hand insertion technique of S2 sacral alar‐iliac screws for spino‐pelvic fixation: technical note, acadaveric study. J Korean Neurosurg Soc. 2015;58:578–81. 26819698 10.3340/jkns.2015.58.6.578 PMC4728101

[14] Park YS , Hyun SJ , Park JH , Kim KJ , Jahng TA , Kim HJ . Radiographic and clinical results of freehand S2 alar‐iliac screw placement for spinopelvic fixation: an analysis of 45 consecutive screws. Clin Spine Surg. 2017;30:E877–82. 28234776 10.1097/BSD.0000000000000520

[15] Fang T , Russo GS , Schroeder GD , Kepler CK . The accurate free‐hand placement of S2 alar iliac (S2AI) screw. Clin Spine Surg. 2020;33:102–3. 29553943 10.1097/BSD.0000000000000623

[16] Choi HY , Hyun SJ , Kim KJ , Jahng TA , Kim HJ . Freehand S2 alar‐iliac screw placement using K‐wire and cannulated screw: technical case series. J Korean Neurosurg Soc. 2018;61(1):75–80. 29354238 10.3340/jkns.2016.1212.008 PMC5769852

[17] Oh CH , Yoon SH , Kim YJ , Hyun D , Park HC . Technical report of free hand pedicle screw placement using the entry points with junction of proximal edge of transverse process and lamina in lumbar spine: analysis of 2601 consecutive screws. Korean J Spine. 2013;10(1):7–13. 24757450 10.14245/kjs.2013.10.1.7 PMC3941737

[18] Hu X , Ohnmeiss DD , Lieberman IH . Robotic‐guided sacro‐pelvic fixation using S2 alar‐iliac screws: feasibility and accuracy. Eur Spine J. 2017;26:720–5. 27272491 10.1007/s00586-016-4639-5

[19] Laratta JL , Shillingford JN , Lombardi JM , Alrabaa RG , Benkli B , Fischer C , et al. Accuracy of S2 alar‐iliac screw placement under robotic guidance. Spine Deform. 2018;6:130–6. 29413734 10.1016/j.jspd.2017.08.009

[20] Ray WZ , Ravindra VM , Schmidt MH , Dailey AT . Stereotactic navigation with the O‐arm for placement of S‐2 alar iliac screws in pelvic lumbar fixation. J Neurosurg Spine. 2013;18:490–5. 23495892 10.3171/2013.2.SPINE12813

[21] Bederman SS , Hahn P , Colin V , Kiester PD , Bhatia NN . Robotic guidance for S2‐alar‐iliac screws in spinal deformity correction. Clin Spine Surg. 2017;30:E49–53. 28107243 10.1097/BSD.0b013e3182a3572b

[22] El Dafrawy MH , Raad M , Okafor L , Kebaish KM . Sacropelvic fixation: a comprehensive review. Spine Deform. 2019;7:509–16. 31202365 10.1016/j.jspd.2018.11.009

[23] O'Brien JR , Yu WD , Bhatnagar R , Sponseller P , Kebaish KM . An anatomic study of the S2 iliac technique for lumbopelvic screw placement. Spine. 2009;34:E439–42. 19454996 10.1097/BRS.0b013e3181a4e3e4

[24] Lin JD , Tan LA , Wei C , Shillingford JN , Laratta JL , Lombardi JM , et al. The posterior superior iliac spine and sacral laminar slope key anatomical landmarks for freehand S2‐alar‐iliac screw placement. J Neurosurg Spine. 2018;29:429–34. 30052147 10.3171/2018.3.SPINE171374

[25] Shillingford JN , Laratta JL , Park PJ , Lombardi JM , Tuchman A , Saifi C , et al. Human versus robot: a propensity‐matched analysis of the accuracy of free hand versus robotic guidance for placement of S2 alar‐iliac (S2AI) screws. Spine. 2018;43:E1297–304. 29672421 10.1097/BRS.0000000000002694

[26] Sponseller PD , Zimmerman RM , Ko PS , Pull ter Gunne AF , Mohamed AS , Chang TL , et al. Low profile pelvic fixation with the sacral alar iliac technique in the pediatric population improves results at two⁃year minimum follow⁃up. Spine. 2010;35:1887–92. 20802390 10.1097/BRS.0b013e3181e03881

[27] Li J , Hu Z , Tseng C , Zhao Z , Yuan Y , Zhu Z , et al. Radiographic and clinical outcomes of surgical correction of poliomyelitis‐related spinal deformities: a comparison among three types of pelvic instrumentations. World Neurosurg. 2019;122:e1111–9. 30439526 10.1016/j.wneu.2018.10.238

[28] Elder BD , Ishida W , Lo SL , et al. Use of S2‐alar‐iliac screws associated with less complications than iliac screws in adult lumbosacropelvic fixation. Spine. 2017;42:E142–9. 27254657 10.1097/BRS.0000000000001722

[29] Ilyas H , Place H , Puryear A . A comparison of early clinical and radiographic complications of iliac screw fixation versus S2 alar iliac (S2AI) fixation in the adult and pediatric populations. J Spinal Disord Tech. 2015;28:E199–205. 25627809 10.1097/BSD.0000000000000222

[30] Mazur MD , Mahan MA , Shah LM , Dailey AT . Fate of S2‐alar‐iliac screws after 12‐month minimum radiographic follow‐up: preliminary results. Neurosurgery. 2017;80:67–72. 27341341 10.1227/NEU.0000000000001322

[31] Smith EJ , Kyhos J , Dolitsky R , Yu W , O'Brien J . S2 alar iliac fixation in long segment constructs, a two‐ to five‐year follow‐up. Spine Deform. 2018;6:72–8. 29287821 10.1016/j.jspd.2017.05.004

[32] Keorochana G , Arirachakaran A , Setrkraising K , Kongtharvonskul J . Comparison of complications and revisions after sacral 2 alar iliac screw and iliac screw fixation for Sacropelvic fixation in pediatric and adult populations systematic review and meta‐analysis. World Neurosurg. 2019;132:408–20. 31465853 10.1016/j.wneu.2019.08.104

[33] Hasan MY , Liu G , Wong HK , Hao TJ . Post‐operative complications of S2AI versus iliac screw in spinopelvic fixation a meta‐analysis and recent trends review. Spine J. 2019;20:964–72. 31830594 10.1016/j.spinee.2019.11.014

[34] De la Garza RR , Nakhla J , Sciubba DM , Yassari R . Iliac screw versus S2 alar‐iliac screw fixation in adults: a meta‐analysis. J Neurosurg Spine. 2018;30:253–8. 30497149 10.3171/2018.7.SPINE18710

[35] Guler UO , Cetin E , Yaman O , et al. Sacropelvic fixation in adult spinal deformity (ASD); a very high rate of mechanical failure. Eur Spine J. 2015;24:1085–91. 25323138 10.1007/s00586-014-3615-1

[36] Ishida W , Elder BD , Holmes C , Goodwin CR , Lo SFL , Kosztowski TA , et al. S2‐alar‐iliac screws are associated with lower rate of symptomatic screw prominence than iliac screws radiographic analysis of minimal distance from screw head to skin. World Neurosurg. 2016;93:253–60. 27319308 10.1016/j.wneu.2016.06.042

